# FRET‐Integrated Polymer Brushes for Spatially Resolved Sensing of Changes in Polymer Conformation

**DOI:** 10.1002/anie.202104204

**Published:** 2021-06-17

**Authors:** Quinn A. Besford, Huaisong Yong, Holger Merlitz, Andrew J. Christofferson, Jens‐Uwe Sommer, Petra Uhlmann, Andreas Fery

**Affiliations:** ^1^ Institute of Physical Chemistry and Polymer Physics Leibniz-Institut für Polymerforschung e. V. Hohe Str. 6 01069 Dresden Germany; ^2^ Institute Theory of Polymers Leibniz-Institut für Polymerforschung e. V. Hohe Str. 6 01069 Dresden Germany; ^3^ School of Science RMIT University Melbourne Victoria 3001 Australia

**Keywords:** chemosensing, fluorescence, FRET, polymer brushes, polymer dynamics

## Abstract

Polymer brush surfaces that alter their physical properties in response to chemical stimuli have the capacity to be used as new surface‐based sensing materials. For such surfaces, detecting the polymer conformation is key to their sensing capabilities. Herein, we report on FRET‐integrated ultrathin (<70 nm) polymer brush surfaces that exhibit stimuli‐dependent FRET with changing brush conformation. Poly(*N*‐isopropylacrylamide) polymers were chosen due their exceptional sensitivity to liquid mixture compositions and their ability to be assembled into well‐defined polymer brushes. The brush transitions were used to optically sense changes in liquid mixture compositions with high spatial resolution (tens of micrometers), where the FRET coupling allowed for noninvasive observation of brush transitions around complex interfaces with real‐time sensing of the liquid environment. Our methods have the potential to be leveraged towards greater surface‐based sensing capabilities at intricate interfaces.

## Graphical abstract

FRET chemistry was integrated within stimuli‐responsive polymer brush layers on planar substrates for spatial sensing of changing polymer conformations. Sensing was demonstrated for a variety of liquid mixture compositions, including high‐resolution observation of lateral differences in polymer conformation at immiscible liquid interfaces (see picture), thus offering great potential to be leveraged for new surface‐based sensing devices.

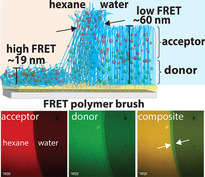

## Introduction

Polymer brush surfaces that have the capacity to switch their physical properties, including wettability,^[1]^ adhesion,^[2]^ lubrication,^[3]^ and exposed surface groups,^[4]^ hold great potential for use in the next generation of sensing materials. Polymer brushes consist of thin films of polymer chains covalently tethered to surfaces with a small grafting distance compared to their respective radii of gyration in solution.^[5]^ These surface layers exhibit functionality when stimuli cause the polymer chains to undergo a phase transition.^[6]^ The stimuli can come in many forms that are unique to each polymer, but can include temperature,^[7]^ or isothermally by solvent,^[8]^ pH,^[9]^ and ionic strength,^[10]^ which can be coupled together for multimodal sensing capabilities.^[11]^ The phase transitions result in an ensemble change in the conformation of the polymer chains, from the extremes of collapsed to fully extended (i.e., swollen). These transitions are dramatic as the high grafting density and uniformity in chain height throughout the brush results in rapid and communal switching transitions.^[12]^ The response can be tailored by precise control over chemical and structural parameters, such as the brush thickness, density and architecture.^[13]^ Importantly, by harnessing this power of phase transitions it is possible to extrapolate signals corresponding to stimuli, thereby allowing for surface‐based sensing of environmental conditions. For planar polymer brush systems, most conventional analyses of polymer brush conformation rely on ex situ methods such as atomic force microscopy (AFM), spectroscopic ellipsometry, and/or quartz crystal microbalance.^[14]^ These methods have restricted spatial resolution, where they are unable to spatially resolve and sense processes that occur around complex architectures (e.g., at surfaces that are “wet” by liquid–liquid interfaces) or to analyse conformational states in constrained geometries, such as in nanopores. To overcome these challenges in spatially sensing conformational changes, engineering new signal transduction mechanisms that function on polymer brush height, are being pursued for greater surface‐based sensing capabilities to be achieved.^[15]^

Towards this goal, recent works have focused on incorporating functional motifs spatially within polymer brushes that amplify and transduce signals from changing brush conformations. This has included incorporating plasmonic nanoparticles into polymer brush structures that allow for UV–visible spectroscopy (UV/Vis) monitoring of plasmonic band shifts due to particle–particle proximity in the collapsing brush.[^14^, ^16^] Alternatively, luminescent nanocrystals^[17]^ or molecular fluorophores^[18]^ can be integrated into responsive polymer brush layers for selective fluorescence emission intensity that depends on polymer conformation. Tas et al., reported on polymer brush systems with end‐tethered fluorophores, where the fluorophore exhibited quenching effects which were correlated to the polymer brush height.^[19]^ This has separately been used for microscopically visualizing patterns.^[20]^ These fluorescence‐based approaches offer strong possibilities towards developing more complex polymer brush surfaces that can noninvasively reveal details on polymer conformation, and ultimately sensing of real‐time changes in aqueous solution compositions.

Herein, we introduce Förster resonance energy transfer (FRET) chemistry into ultrathin (<70 nm) poly(*N*‐isopropyl acrylamide) (PNIPAM) polymer brush surfaces for use as in‐liquid optical sensors of polymer conformation under different stimuli. The PNIPAM was chosen due to its exceptional sensitivity to various stimuli, including subtle changes in liquid mixture compositions (co‐nonsolvency effects—polymer collapse at intermediate mixing ratios of two good solvents^[21]^), and their ability to be assembled into well‐defined polymer brushes. The polymer chains were assembled into dense and homogenously smooth polymer brushes on planar quartz surfaces by a grafting‐to approach. The surfaces exhibited strong fluorescence, with a brush thickness in water of approximately 65 nm. When incubated in mixtures that give rise to co‐nonsolvency effects, FRET pairing occurred as the polymer brush collapsed, then FRET decreased as the surfaces were immersed in “good” solvent systems. This process allowed for high‐resolution monitoring of lateral changes in surface polymer brush conformation. Importantly, our approach is not limited to PNIPAM systems, where we anticipate other functional polymers can be used in a similar way, leading to enhanced in‐liquid sensing capabilities.

## Results and Discussion

**Figure 1:**
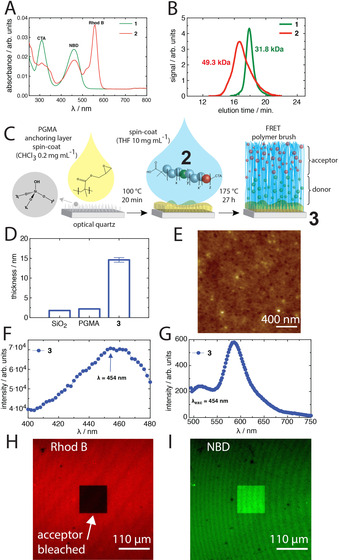
A) UV/Vis spectra (1 mg mL^−1^ in EtOH) and B) GPC traces of **1** (*Ð*=1.13) and **2** (*Ð*=1.55). C) Assembly of **2** as polymer brushes on poly(glycidyl methacrylate)(PGMA)‐coated optical quartz substrates. D) Thickness of the native SiO_2_ surface (ca. 1.7 nm), the PGMA layer and the dry polymer brush. E) Dry‐state AFM image. F) Fluorescence excitation spectra. G) Emission spectra. H, I) CLSM images of acceptor (Rhod B) (H) and donor (NBD) channels (I) after a square region was photobleached at 543 nm (acceptor).

**Scheme 1:**
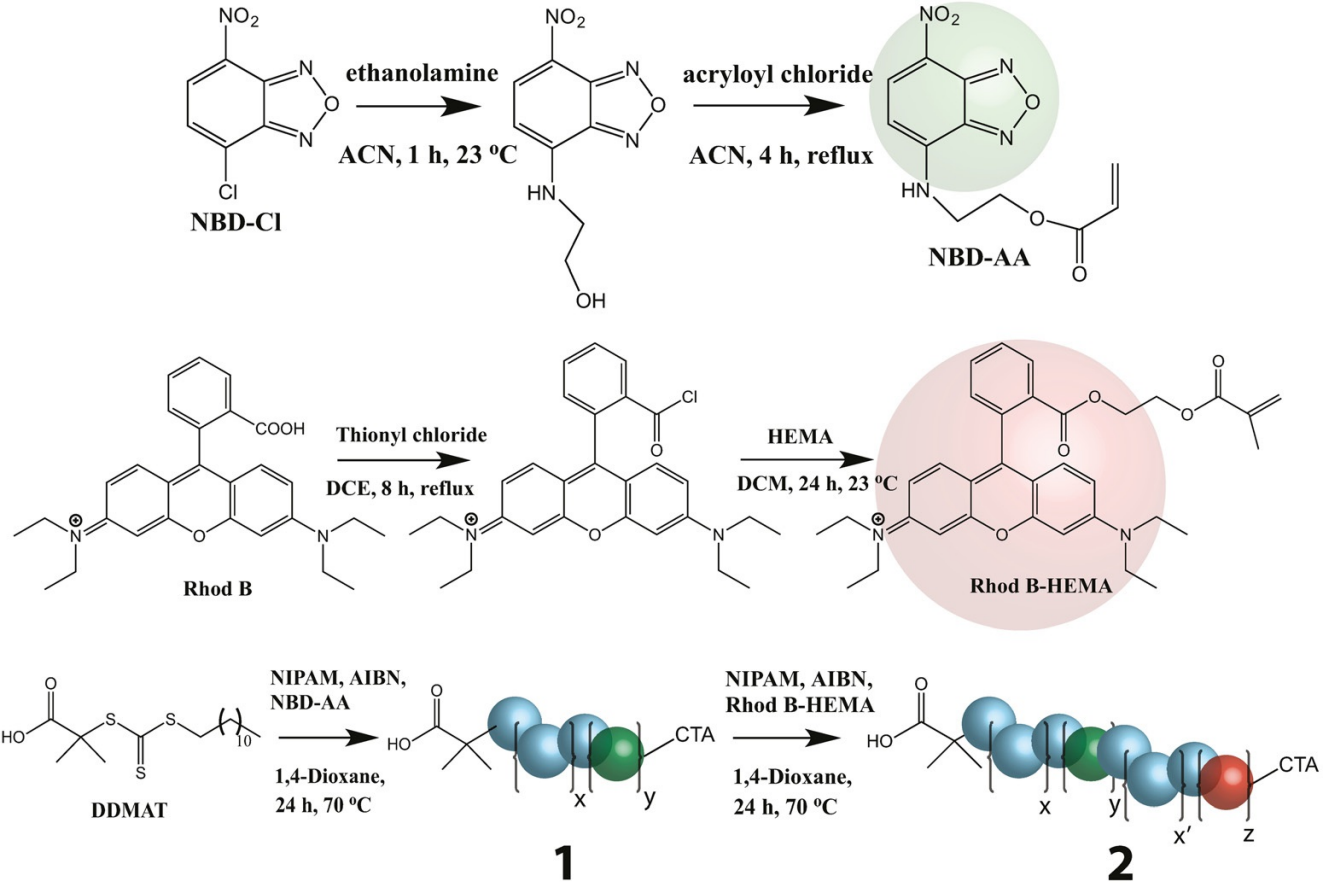
Synthesis of donor (NBD‐AA) and acceptor (Rhod B‐HEMA) monomers, and their combination as a diblock random copolymer **2** with NIPAM. ACN, acetonitrile; NBD‐Cl, 4‐chloro‐7‐nitrobenzo‐2‐oxa‐1,3‐diazole; NBD‐AA, 4‐(2‐aminoethyl acrylate)‐7‐nitrobenzo‐2‐oxa‐1,3‐diazole; DCE, dichloroethane; DCM, dichloromethane; HEMA, 2‐hydroxyethyl methacrylate; NIPAM, *N*‐isopropylacrylamide; CTA, chain transfer agent.

Towards integrating FRET chemistry within functional PNIPAM polymers, we synthesised a FRET donor monomer composed of 4‐(2‐acryloyloxyethylamino)‐7‐nitro‐2,1,3‐benzoxadiazole (NBD) (Scheme 1), and a FRET acceptor monomer composed of rhodamine B (Rhod B). This combination of FRET probes have been used in previous studies of single‐chain temperature and pH sensors.^[22]^ ^1^H NMR confirmed the successful synthesis of the NBD monomer, NBD‐AA, and the Rhod B monomer, Rhod B‐HEMA (Figure S1). Polymers were synthesised by reversible addition–fragmentation chain‐transfer (RAFT) polymerisation from the chain transfer agent (CTA) 2‐(dodecylthiocarbonothioylthio)‐2‐methylpropionic acid (DDMAT), with azobisisobutyronitrile (AIBN) as the initiator. The use of DDMAT was purposefully chosen so as to yield a carboxylate group of the initiating end of the resulting polymers, which was needed for polymer brush assembly. The donor monomer was integrated within the first block with NIPAM, yielding a macroscopic NIPAM/NBD random copolymer‐CTA (product **1**). This purified polymer was subsequently subjected to a second polymerization with Rhod B‐HEMA and NIPAM, yielding the final diblock random copolymer (product **2**). The products were yellow and orange in appearance (Figure S2), respectively. UV/Visible spectroscopy (UV/Vis) of the product **1** showed clear absorption corresponding to NBD (ca. 460 nm), which for product **2** was complemented by a secondary Rhod B absorption (ca. 556 nm; Figure 1 A). Gel permeation chromatography (GPC) revealed product **1** to have a number average molecular weight, *M*_N_, of about 30 kDa, whereas product **2** was about 50 kDa (Figure 1 B). The dispersity, *Ð*, was higher for product **2** (*Ð*=1.55 compared to 1.13), but within an acceptable range, which was likely due to the Rhod B‐HEMA monomer interfering with chain propagation. A UV/Vis standard assay was performed for each monomer and product polymer to determine the amount of each fluorophore incorporated into each polymer chain. It was found that, on average, each chain contained 0.712 monomers of NBD and 1.51 monomers of Rhod B.

Polymer brushes were assembled by a grafting‐to approach that exploits macromolecular anchoring polymers.^[23]^ This process involves first modifying optical quartz surfaces with a thin layer (ca. 2 nm) of poly(glycidyl methacrylate) (PGMA), where surface Si‐OH groups are allowed to conjugate to a fraction of the epoxide groups of PGMA (Figure 1 C). Subsequently, a solution of product **2** was allowed to conjugate to the anchoring PGMA through the carboxylate groups from the initiating DDMAT CTA. The resulting polymer brushes were approximately 15 nm in dry height (Figure 1 D) and exhibited very low surface roughness, with a root‐mean‐square (RMS) roughness of about 434 pm (Figure 1 E). The grafting density was determined from *σ*=(*hρN*_A_)/Mn,^[24]^ where h is the dry brush thickness, and *ρ* the bulk density of the brush composition, which we take as 1.1 g cm^−3^, and N_A_ is Avogadro's number. We found that *σ*≈0.19 chains/nm^2^, consistent with previously reported densely grafted PNIPAM brushes.^[25]^ This emphasises the utility of our macromolecular anchoring approach of carboxylate end‐group PNIPAM polymers, from a melt, for assembling dense PNIPAM brush surfaces. Importantly, the surfaces exhibited fluorescence excitation spectra consistent with NBD's excitation wavelength at 454 nm (Figure 1 F), where the emission showed clear Rhod B emission in the dry state (Figures 1 G and S3), indicating strong FRET pairing. This was confirmed by confocal laser scanning microscopy (CLSM) measurements of the surfaces with both the acceptor channel (width between 560 nm and 700 nm, with *λ*_exc_=543 nm) and donor channel (width between 490 and 560 nm, with *λ*_exc_=458 nm), after a square section was photobleached at *λ*_exc_=543 nm for 40 minutes (Figure 1 H and I, respectively). It was clearly seen that where the acceptor was bleached there was significant enhancement of the donor fluorescence (Figure S4), validating the FRET pairing for the polymer brush surface. The polymer brush surfaces have the FRET donor in closest proximity to the quartz, with the Rhod B extended outwards finishing with the CTA on the solvent side. This architecture ensures changes in FRET pairing should be reflective of both the polymer brush height, and the individual mixing of chains amongst their neighbours (i.e., a multidimensional probe of the polymer conformation).

These surfaces were investigated under co‐nonsolvency conditions, which causes PNIPAM to collapse at intermediate mixing ratios of two “good” solvents.^[25]^ Common co‐nonsolvency systems for PNIPAM include mixtures of short‐chain alcohols with water. This effect was pursued as it offers an intriguing possibility for chemosensing of small compositional changes in aqueous liquids that causes dramatic changes in PNIPAM conformation. The PNIPAM therefore provides an excellent test system to probe and sense stimuli‐induced changes in polymer conformation.

**Figure 2:**
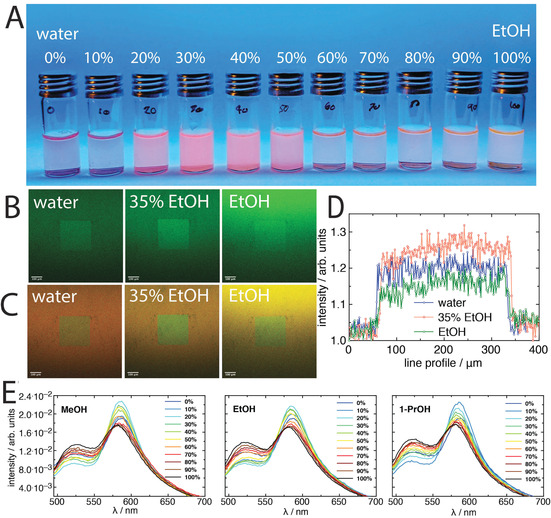
A) A digital photograph of solutions containing free polymer **2** dissolved in various water/ethanol mixtures under UVA light (10 mg mL^−1^). B, C) CLSM images of the donor (*λ*_exc_=458 nm) (B) and composite channel (C) of the acceptor photobleached regions in different water/EtOH solutions, along with D) the corresponding intensity profile across the photobleached square of the donor channel, which was normalised by the intensity at 0 μm. E) Fluorescence emission spectra of the polymer brush surfaces **3** in different alcohol/water mixtures. For all spectra *λ*_exc_=454 nm, and for all CLSM images the scale bar is 100 μm; all measurements were performed at 24 °C.

When the free polymer, product **2**, was incubated in a series of mixtures spanning pure water to pure ethanol, EtOH, it was found that differences could be seen visually of both the turbidity and colour of the solutions (Figure 2 A), indicating phase separation of the polymer in co‐nonsolvency conditions. The pink colour of the solutions with approximately 20–50 % EtOH indicates that the Rhod B exhibits greater fluorescence (i.e., FRET pairing during chain collapse) under co‐nonsolvency conditions. The behaviour of the polymer brush (product **3**) in co‐nonsolvency conditions was investigated by CLSM, where an acceptor photobleached region showed different contrast from extended brush conformations to collapsed for both the FRET donor channel and composite images (Figure 2 B and C, respectively). This is reflective of the polymer brush that is surrounding the photobleached region extending in conformation (i.e., less FRET=less contrast), and collapsing under co‐nonsolvency (i.e., more FRET=greater contrast). This was clarified by line profile analysis across the photobleached region (Figure 2 D), where a greater intensity was observed for the collapsed region (FRET occurring, which removes donor intensity from the area surrounding the photobleached square). This intensity, with respect to the surrounding area, then decreased in the presence of “good” solvents (35 % EtOH>water>EtOH).^[21]^ The photobleaching therefore offers a potential way of spatially visualising polymer brush conformation on photobleached patterns. The fluorescence spectra of the polymer brush surfaces in different aqueous mixtures of water and methanol (MeOH), EtOH, and 1‐propanol (1‐PrOH), were characterised separately by fluorescence spectroscopy (Figure 2 E), all with *λ*_exc_=454 nm. It was found that significant FRET pairing occurs in co‐nonsolvency mixtures, where the chains are expected to collapse.^[26]^ The polymer brush FRET (Figure 2 E) qualitatively matches the pairing seen for the free polymer systems (i.e., greater FRET under co‐nonsolvency conditions; Figure S5), however the magnitude of the change in FRET was found to be lower for the brush in comparison to the free polymer. This difference in the magnitude of the FRET can be understood to result from the proximity of donor and acceptor pairs between neighbouring chains in the polymer brush, where the distance of separation between FRET pairs is not only a function of the single chain, but also of the separation between all pairs between neighbouring chains that mix together. Whereas for single chains in a dilute system all FRET occurrences should result from single‐chain interactions, rather than neighbouring interactions.

**Figure 3:**
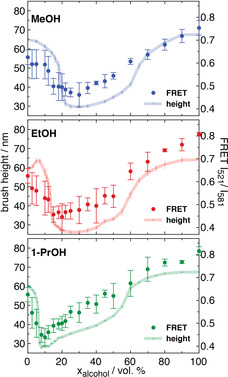
A) Comparison of the FRET pairing (I_521_/I_581_) for the polymer brush systems (right axes) in aqueous water–alcohol mixtures, along with ellipsometry measurements of the polymer brush height for the same systems (left axes). Ellipsometry measurements were performed at 24 °C.

The FRET pairing was analysed further with respect to the ratio of the FRET donor‐to‐acceptor peaks, to the polymer brush height as measured by ellipsometry. This was important to directly gauge the effects of FRET directly against polymer conformation. For the FRET ratio, the donor was taken as the intensity at 521 nm, I_521_, and the acceptor at 581 nm, I_581_. For the case of single free polymer chains, when I_521_/I_581_≫1, the chains are extended, whereas when I_521_/I_581_≪1 the chains are collapsed. For the polymer brush, the magnitude of the differences in I_521_/I_581_ are expected to be shifted due to a degree of permanent FRET that occurs between chains, but the trend in the ratios should be similar. It was found that for the FRET, typical co‐nonsolvency transitions were exhibited (i.e., FRET ratios consistent with collapsed polymers at intermediate volume fractions of alcohol in water^[21]^). Particularly, the onset of FRET was at greater volume fractions of alcohol for MeOH>EtOH>1‐PrOH (Figure 3), which matches known co‐nonsolvency trends. Crucially, by directly comparing to polymer brush height, there were clear similarities between the onset of polymer brush collapse for each system, thereby showing congruence between FRET and polymer brush height. However, there were some notable differences regarding the re‐extension of the brushes. Typically, in alcohol‐rich mixing conditions, the polymer brushes are slightly more extended in height in comparison to pure water. However, we find that for the FRET pairing, this is more significantly extended (distance between donor and acceptor monomers) for the same conditions. This likely highlights the capability of the FRET pairing to provide further insight into the mixing of chains within the polymer brush (i.e., the extension and collapse that is not purely in the perpendicular direction to the surface), rather than only polymer brush height, as probed by ellipsometry.

Whilst the FRET is consistent with polymer brush height under co‐nonsolvency conditions, it is important to verify that solvatochromic effects (i.e., different donor fluorescence output dependent on solvent) in the different solvent systems are not hampering our interpretation of the FRET. This was first investigated by performing time‐dependent density functional theory (TD‐DFT) calculations to determine the oscillator strengths of optimised NBD and Rhod B dye structures in pure water and methanol. It was found that both dyes have similar oscillator strengths in these solvents (Table S1), thereby suggesting that solvatochromic effects are not significant in our FRET analysis.

**Figure 4:**
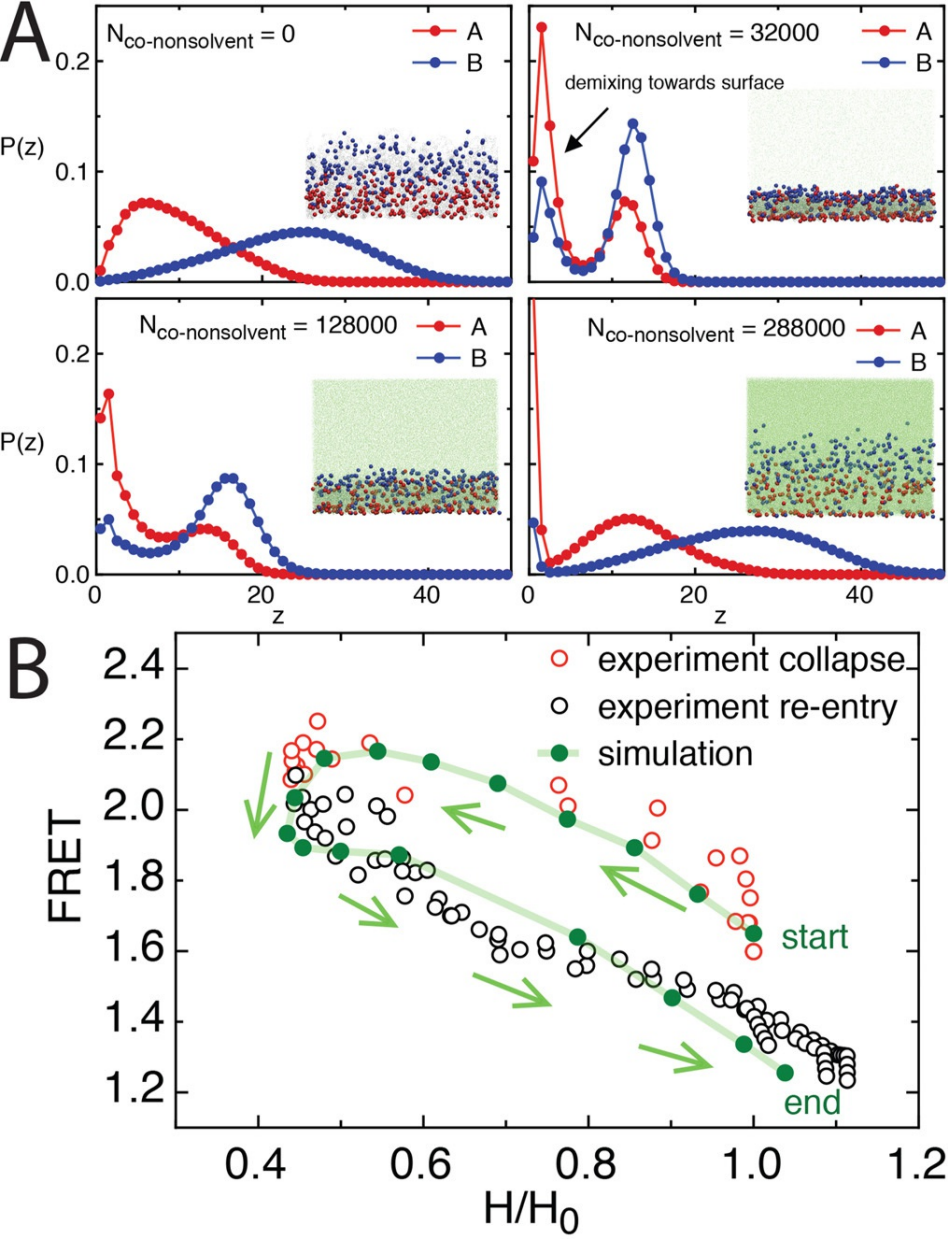
A) Simulated distributions of A‐ and B‐type monomers in the perpendicular dimension (*z*) in the model polymer brush under different co‐nonsolvency conditions. When the number of co‐nonsolvent molecules, N_co‐nonsolvent_=0, the brush is swollen; N_co‐nonsolvent_=32 000, the brush is fully collapsed; N_co‐nonsolvent_=128000, the brush is re‐entering extended state; N_co‐nonsolvent_=288 000, the brush is re‐swollen. The substrate is situated at *z=*0, and a fraction of chromophores show partial demixing at the substrate. All densities are normalised so that $\int P\left(z\right)dz=1$ , and *z* is given in multiples of the bead‐diameter (see the Supporting Information for further details. Inserts show snapshots of the corresponding trajectories, where A‐ and B‐type monomers (chromophores) are rendered red and blue, respectively, with their actual diameters, and other monomers are rendered as grey, and the cosolvent as green. B) Comparison of the experimental FRET success (here given as I_581_/I_521_) to polymer brush height throughout the collapse and re‐entry transitions, along with data from the simulated systems. The height of the polymer brush, H, was normalised by its reference value without cosolvent, H_0_.

This was further validated by performing molecular dynamics simulations of model diblock polymer brushes that have pseudo donor and acceptor monomers in each block. The simulated FRET was then estimated from the distance between donor and acceptor monomers with a long‐range cut‐off, which would reflect the distance dependence of real FRET (Figure 4 A). Full details are provided in the Supporting Information. The simulated FRET can then be directly compared to the simulated polymer brush height, as well as to the experimental FRET and height, in order to examine the FRET relation. It was found that there is a clear separation between the experimental FRET vs. height during the polymer brush collapse and re‐entry transitions (Figure 4 B). Importantly, this relation was strongly matched by the simulated system that does not have solvatochromic effects, thereby verifying our interpretation of the FRET results. Furthermore, the information in Figure 4 reveals further details on the transitions of the polymer brush that are not readily accessible by AFM or ellipsometry methods, which highlights the capability of the FRET pairing to provide further insight into the mixing of chains within the polymer brush (i.e., the extension and collapse that is not only in the perpendicular direction to the surface). Interestingly, the inclusion of the fluorophores within the polymer brush has not significantly varied the co‐nonsolvency transitions of PNIPAM that has been found for homopolymer systems,^[21]^ though we do note a small degree of demixing of the simulated fluorophores against the surface under co‐nonsolvency conditions (Figure 4 A). This occurred due to the co‐nonsolvent interacting more strongly with the polymer backbone than with the donor and acceptor monomers, leading to a partial demixing of these monomers against the substrate when the concentration of co‐nonsolvent molecules was high. However, broadly speaking, our strategy retains the chemosensitivity of native PNIPAM whilst allowing for incorporation of complex optical functionalities.

**Figure 5:**
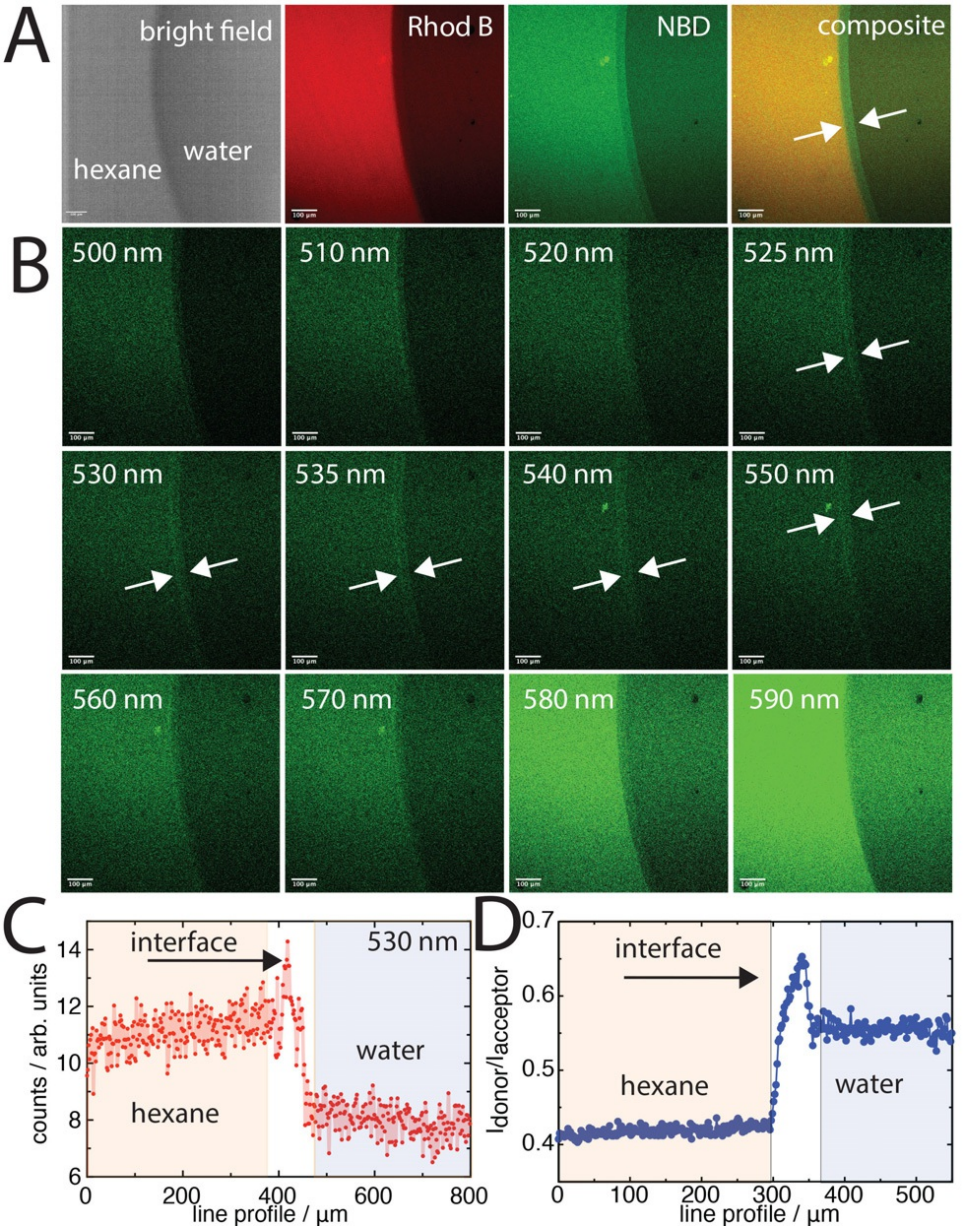
A) Bright‐field and CLSM images of the interface between hexane and water, along with B) lambda images of stepwise pixel emission in the range of 500–590 nm (increased emission at the interface indicated by arrows). The images represent intensities of emission per pixel at each wavelength indicated in the top‐left corner from excitation at 458 nm (i.e., not donor or acceptor channels). C) Line profile the counts in the 530 nm profile. D) FRET ratio of the donor channel to acceptor channel across the interfacial region. For (C) and (D) the regions are coloured schematically to guide the eye. For all CLSM images the scale bar is 100 μm; all measurements were performed at 24 °C.

The use of FRET with CLSM offers the unique capability to probe spatial changes in polymer brush conformation. This was investigated by CLSM imaging of the interface of a water droplet within hexane (Figure 5 A) across the FRET channels. It was observed that in the hexane phase the polymer brush exhibited a greater fluorescence intensity over both channels. This greater intensity in the hexane phase was attributed to aggregation‐induced emission, where the polymer brush was significantly more collapsed (ca. 19±3.4 nm) than under the co‐nonsolvency conditions (Figure 3), however this does not affect the FRET ratio analysis (i.e., both channels exhibit increased intensity). We found that at the hexane–water interface, the FRET donor had greater emission (FRET decoupling), allowing for spatial visualisation of a clear region of difference in the composite donor/acceptor image. This was investigated further by Lambda imaging of the interface, where the emission profile of each pixel was collected over the entire image. We found that a clear region of FRET donor intensity emerges at the interface between the emission of about 525–550 nm (Figure 5 B,C). The total fluorescence profile over the whole image indicated that the FRET occurred mostly in the hexane phase (collapsed; brush height ≈19±3.4 nm), which decreased in the water phase (transition towards extended; brush height ≈61±1.1 nm). However, at the interface itself the FRET was mostly decreased (Figure 5 D), where the intensity of NBD was greater, which indicates that the polymer brush is extended. This region spanned approximately 40 μm across the interfacial region. Interestingly, this may indicate the “pulling” of the polymer brush at the pinned interface towards the preferred phase (i.e., water), rather than simply collapsing. Our method therefore allows for more detailed spatial information on the polymer brush dynamics in such systems that is not directly accessible by other methods.

Lastly, our strategy for spatially extrapolating signals from polymer brush transitions is not limited to planar geometries. Through adjustment of the grafting‐to procedure, we expect it will be possible to spatially sense conformational changes around more complex macroscopic architectures, including in microfluidic devices^[27]^ and on patterned and wrinkled surfaces.^[28]^ We anticipate that greater sensitivity may be achieved by exploring different chain architectures for the distribution of donor and acceptor monomers, and also the inclusion of cascade FRET (an additional longer wavelength acceptor monomer). Furthermore, the polymer backbone of our brushes is not limited to PNIPAM, where further functionalities can be explored. However, the PNIPAM may allow for further investigation of co‐nonsolvency transitions at complex aqueous interfaces, such as for studying Criegee intermediates^[29]^ and ethanol release from yeast cells.

## Conclusion

In conclusion, we have reported on FRET‐integrated planar polymer brush surfaces that provide FRET output depending on the conformation of the polymers in liquid mixtures. The FRET output was used as a basis to extrapolate sensing information from the conformational transitions of the polymer brush, which for the studied PNIPAM has a strong dependency on the nature of the liquid mixture, thereby providing optical sensing of the solvating liquid compositions. Our FRET‐integrated surfaces have the capacity to allow for greater in‐liquid surface‐based sensing capabilities to be realised, especially around complex interfaces.

## Conflict of interest

The authors declare no conflict of interest.

## Supporting information

As a service to our authors and readers, this journal provides supporting information supplied by the authors. Such materials are peer reviewed and may be re‐organized for online delivery, but are not copy‐edited or typeset. Technical support issues arising from supporting information (other than missing files) should be addressed to the authors.

Supplementary
