# Supplementary material for: FRET‐Integrated Polymer Brushes for Spatially Resolved Sensing of Changes in Polymer Conformation
Source: Angew Chem Int Ed Engl. 2021 Jun 17;60(30):16600–6. doi: 10.1002/anie.202104204 (PMC8361709; doi:10.1002/anie.202104204)
Supplement: Supplementary file 1 — Supplementary [file ANIE-60-16600-s001.pdf]

Angewandte Chemie
Eine Zeitschrift der Gesellschaft Deutscher Chemiker

Supporting Information

FRET-Integrated Polymer Brushes for Spatially Resolved Sensing of Changes in Polymer Conformation

Quinn A. Besford, Huaisong Yong, Holger Merlitz, Andrew J. Christofferson, Jens-Uwe Sommer, Petra Uhlmann, and Andreas Fery**

anie_202104204_sm_miscellaneous_information.pdf

Supporting Information
Table of Contents

Experimental Procedures

**Materials**	**3**
**Synthesis**	**3**
**Formation of polymer brushes**	**4**
**Instrumental details**	**4**
**Simulations**	**5**
**TD-DFT**	**6**

Results and Discussion

**^{1}H NMR spectra**	**7**
**Product appearance**	**8**
**CLSM**	**8**
**Fluorescence spectra**	**9**
**TD-DFT**	**10**

Experimental Procedures

Materials. All chemicals where of analytical grade and used as received without purification, with the exception of 2-hydroxyethyl methacrylate (HEMA) and *N*-isopropylacrylamide (NIPAM) which were purified by passing through an aluminium oxide column prior to reaction, and by recrystallisation from hexane (× 2), respectively, to remove inhibitors. High-purity water (Milli-Q water) with a resistivity of >18.2 MΩ cm was obtained from an inline Millipore RiOs/Origin water purification system (Millipore Corporation, Massachusetts, USA). Polished single-crystal (100)-silicon wafers were obtained from Silicon Materials, Kaufering, Germany, with a native SiO_2 layer thickness of ~1.7 nm. Optical fused quartz square cover slips (22 × 22 × 0.2 mm) were obtained from Micro to Nano (Haarlem, The Netherlands). PGMA (M_N = 15 000 g mol^{-1}, Đ = 1.6) was obtained from Polymer Source Inc. (Montreal, Canada). 4-Chloro-7-nitrobenzofurazan (NBD-Cl), ethanolamine, acetonitrile (ACN), acryloyl chloride, thionyl chloride, dichloroethane (DCE), rhodamine B (Rhod B), HEMA, dichloromethane (DCM), NIPAM, azobisisobutynitrile (AIBN), 1,4-dioxane (anhydrous), tetrahydrofuran (THF), diethyl ether, hexane, methanol, ethanol, and 1-propanol were obtained from Sigma-Aldrich. THF was obtained from Acros Organics. Chloroform was obtained from Fisher Chemicals. $CDCl_3$ and DMSO-d_6 was obtained from Eurisotop (Saint-Aubin, France).

Synthesis of 4-(2-hydroxyethylamino)-7-nitro-2,1,3-benzoxadiazole. NBD-Cl (1.5 mmol, 300 mg) was dissolved in ACN (35 mL) with stirring, to which ethanolamine (1.5 mmol, 92 mg) was added and the reaction stirred for 1.5 h. Solvent was then extracted under reduced pressure and the crude product was purified by column chromatography (DCM:MeOH, 30:1, v:v) to yield orange crystals (156 mg, 47% yield). ^{1}H NMR (400 MHz, DMSO-d_6, 25 ºC): δ (ppm) 9.40 (1H), 8.50 (1H), 6.45 (1H), 4.95 (1H), 3.65 (2H), 3.55 (2H) (Figure S1A).

Synthesis of NBD-AA. 4-(2-hydroxyethylamino)-7-nitro-2,1,3-benzoxadiazole (0.56 mmol, 125 mg) was dissolved in ACN (60 mL) with stirring, to which acryloyl chloride (50 mmol, 4 mL) was added dropwise. The reaction mixture was then refluxed for 4 h with rigorous stirring. The solvent was then removed under reduced pressure and the crude product purified by column chromatography (DCM as eluent), to yield an orange powder (110 mg, 71% yield). ^{1}H NMR (400 MHz, DMSO-d_6, 25 ºC): δ (ppm) 8.43 (1H, d), 6.42 (1H, d), 6.18 (1H, d), 6.09 (1H, dd), 5.86 (1H, d), 4.47 (2H, t), 3.75 (2H, m) (Figure S1B).

Synthesis of Rhod B-HEMA. Rhodamine B (2.1 mmol, 1 g) was dissolved in DCE (18 mL), to which thionyl chloride (12.7 mmol, 921 µL) was added dropwise at 23 °C with stirring. After 30 minutes the system was then refluxed for 12 h. The solvent was subsequently removed under reduced pressure and the crude product was redissolved in DCM (15 mL), to which HEMA (9.6 mmol, 1.16 mL) was added dropwise and the system was left stirring overnight at 23 °C. The solvent was subsequently removed under reduced pressure and the crude product purified by column chromatography (DCM:MeOH, 30:1) to yield a red sticky solid (386 mg, 33.2% yield). ^{1}H NMR (400 MHz, DMSO-d_6, 25 ºC): δ (ppm) 8.31 (1H, t). 7.86 (1H, m), 7.76 (1H, t), 7.37 (1H, t), 7.08 (1H, d), 6.97 (1H, d), 6.84 (1H, s), 6.15 (1H, d), 5.15 (1H, m), 4.25 (4H, m), 3.65 (2H, m), 3.35 (4H, m), 1.93 (3H, m), 1.34 (12H, m) (Figure S1C).

Synthesis of RAFT polymer 1. NIPAM (30.4 mmol, 3.53 g), AIBN (0.0152 mmol, 2.5 mg), NBD-AA (0.154 mmol, 42.9 mg), and DDMAT (0.154 mmol, 56 mg) were added to a 15 mL Schlenk tube equipped with a stirrer bar, to which anhydrous 1,4-dioxane (6 mL) was added. The mixture was then degassed by bubbling argon for 1.5 h. Polymerisation was initiated by heating the mixture to 70 ºC with rigorous stirring. The flask was protected from light and allowed to react overnight. After 16 h, the mixture was then exposed to air, and the solvent as removed under reduced pressure. The crude polymer was then re-dissolved in minimal THF before being precipitated into cold diethyl ether (2 × 250 mL), and dried overnight in a vacuum oven at 40 ºC. The product was isolated as a bright yellow powder (2.8 g, 79.3% mass yield).

Synthesis of RAFT polymer 2. The macro-CTA product **1** (0.0285 mmol, 35.1 kDa, 1 g), NIPAM (8.76 mmol, 0.990 g), Rhod B-HEMA (0.0285 mmol, 12.3 mg), and AIBN (0.00285 mmol, 0.47 mg) were dissolved in anhydrous 1,4-dioxane (6 mL) with stirring. The polymerisation and purification was identical to that of product **1**. The product was isolated as a deep orange powder (1.76 g, 88.4% mass yield).

Formation of polymer brushes. Silicon wafers (20 × 13 mm) or fused quartz cover slips (22 × 22 × 0.2 mm) were treated with EtOH in an ultrasonic bath for 20 min at 37 °C, and then dried under nitrogen. Afterwards, Piranha solution (H_2SO_4: H_2O_2, 3:1) was placed on the top side of the substrates and allowed to react for 2 min. The substrates were then carefully washed with Milli-Q water, then EtOH, and subsequently dried under a stream of nitrogen. The cleaned substrates were then activated in an oxygen plasma for 1 min (Harrick, Plasma Cleaner PDC-002 with Plasma Flo PDC-FMG). In order to obtain an anchoring layer for the subsequent grafting-to process of **2**, a filtered solution of PGMA (80 µL (silicon wafer) or 160 µL (quartz) of a 0.2 mg mL^{-1} solution in chloroform) was spin-coated (Spin150 spin coater, Polos, v = 2000 r min^{-1}, a = 1000 r $(min\ s)^{-1}$, t = 10 s) onto the activated substrates. The PGMA anchoring layer was subsequently annealed at 100 °C in a vacuum oven for 20 min to react the silanol groups of the substrate with a fraction of the epoxy group of the PGMA. The remaining epoxy groups were then used for the subsequent grafting-to process. A filtered solution of **2** (120 µL (silicon wafer) or 240 µL (quartz) of a 11 mg mL^{-1} solution) was subsequently spin-coated on the substrates (Spin150 spin coater, Polos, v = 2000 r min^{-1}, a = 1000 r $(min\ s)^{-1}$, t = 10 s), followed by annealing at 170 °C in a vacuum oven for 18 h. To remove non-covalently bound polymer, the resulting substrates where first immersed in Milli-Q water, then extracted in Milli-Q water overnight, then rinsed with EtOH and dried under a stream of nitrogen.

Fluorescence and UV-Vis measurements. All measurements were performed with a multimode microplate reader (Tecan Spark 10M, Switzerland). The UV-Vis of the free polymer **1** and **2** was performed at a concentration of 1 mg mL^{-1} in EtOH dispersed into a XXX. Fluorescence measurements of the free polymer **2** was performed in 96-well black plates (Molecular Probes, Invitrogen) by first placing 0.5 µL of a 1 mg mL^{-1} solution of **2** into each well to dry. After 10 min, 200 µL of each respective water/alcohol mixture was added into each of the wells, leading to a concentration of **2** of 2.5 µg mL^{-1}, and allowed to mix with gentle agitation. A transparent lid was used to prevent solvent evaporation during the experiment. After a further 10 min, fluorescence spectra were recorded (λ_{exc} = 454 nm, dλ = 2 nm, 495 – 700 nm) whilst the temperature was maintained at 24 °C.

GPC measurements. The measurements were performed on a GPC system containing a Jasco PU-2080 Plus HPLC pump, a Jasco Jetstream column thermostat, equipped with one GRAM precolumn, one GRAM 3000 10 µL 300 x 8 mm and one GRAM 30 10 µL 300

x 8 mm analytical column. Detection was performed with a differential RI detector smartline 2300 from Knauer. The oven temperature was set to 70 °C and the flow was adjusted to 1.0 mL min^{-1}. As eluent Dimethylacetamide (DMAc) with 5 mg mL^{-1} LiBr and 1 vol % water was used. The system was calibrated with PMMA standards in a range of 602 – 2 200 000 g mol^{-1}. All the standards were purchased from POLYMER STANDARD SOLUTIONS and used as received. For measurement the samples were dissolved in the eluent (concentration 2-3 mg mL^{-1}), filtered through a 0.2 µm PTFE syringe filter and afterwards injected to the instrument.

Confocal laser scanning microscopy (CLSM). For spatially-resolved fluorescence studies, a combined setup of an Axio Observer Z.1 inverted microscope with an LSM710 cnofocal laser scanning module (Carl Zeiss Microscpy, Germany) was used. Two objectives were used (10x and 50x). The donor was excited by an argon laser (458 nm) and the acceptor with a helium-neon laser (543 nm). For the Lambda measurements, the emission was detected spectrally resolved (4.4 nm resolution). The pinhole was set to 1 AU.

Atomic force microscopy (AFM). In-air tapping mode AFM was performed with a Nanoscope Dimension D3100 V (Veeco Instruments, U. S. A.), with software analysis by NanoScope Analysis 1.7, and with a phase tip (500 kHz, k = 3-4 N m^{-1}).

Nuclear magnetic resonance (NMR) spectroscopy. The ^{1}H (500.13 MHz) NMR spectra were recorded using an AVANCE III 500 Spectrometer (Bruker, Germany) using either $CDCl_3$ or DMSO-d_6 at 30 °C.

Ellipsometry. The ellipsometry experiments were conducted at the constant temperature of 24 ± 0.1°C. The details of conducting in-situ real-time experiments of spectroscopic ellipsometry are reported in our previous studies, for details please refer to reference [1].

Simulations. We apply a coarse-grained model of the polymers, consisting of freely jointed spherical beads that are connected by finite extensible nonlinear elastic (FENE) bonds.[2] Pair potentials are Lennard-Jones (LJ) potentials,

$$V_{LJ}(r) = 4\epsilon\left[\left(\frac{d}{r}\right)^{12} - \left(\frac{d}{r}\right)^{6} - \left(\frac{d}{r_c}\right)^{12} + \left(\frac{d}{r_c}\right)^{6}\right] \quad (1)$$

where ε defines the interaction strength (in units of k_BT, where k_B is the Boltzmann constant and T the absolute temperature), d the bead diameter (or, in case of two beads of different diameters, their average value), r the distance of separation, and r_c the cut-off distance. A polymer is a bead-spring chain consisting of a backbone of N = 120 beads of diameter d = 1 (defining the length-unit). The chromophores are modelled as side-groups of diameter d_c = 2, attached to backbone monomers at random positions as follows: The donors (species A) are randomly attached to monomers of indices [3,55], while the acceptors (species B) are randomly attached to monomers of indices [66,118]. This choice prevents chromopores from being in too close in a vicinity along the chain contour or to the substrate. To create a brush, 225 chains are end-grafted onto an impenetrable substrate in a Cartesian 15 x 15 pattern at a grafting density σ = 0.025 (chains per unit area). Pair interactions between all monomers and chromophores are truncated at the respective minima of the LJ-potentials, ε = 1 and r_c = d $2^{1/6}$, leaving fully repulsive interaction potentials. Co-solvent molecules are represented as individual beads of diameter d = 1, with interaction potentials that are attractive to the monomers (ε = 1.3 and r_c = 2.5) but repulsive to chromophores. The simulation box is periodic in both lateral directions to mimic an infinitely large brush, while bounded with repulsive LJ-walls at the substrate and the upper boundary to prevent particles from escaping the box, which has a height of 70 units.

The simulations are carried out with the open-source LAMMPS simulation package.[3] The equation of motion of each bead is the Langevin equation,

$$m\frac{d^2r}{dt^2} = -\frac{m}{\tau}\frac{dr}{dt} - \frac{\partial V}{\partial r} + F \qquad (2)$$

Where m is the mass (m = 4 for chromophores and m = 1 for monomers and co-solvent), the momentum relaxation time τ (τ = 2 for chromophores and τ = 1 for monomers and co-solvent), V being the total potential and F the white-noise fluctuation force. The Langevin equation simulates the friction and fluctuation imposed by an implicit solvent. The temperature is chosen to be T = 1 and the time step is dt = 0.0025 LJ-times. Initially, all chains are random walks in the positive half-space above the substrate, and co-solvent beads are randomly distributed throughout the simulation box. In a short pre-run of 100000 steps, overlaps among beads are removed with the help of a gradually increasing repulsive potential. It follows a pre-relaxation of 2 million simulation steps with fully repulsive LJ-potentials, during which the polymer brush is allowed to swell. After that, the attractive interaction between monomers and co-solvent molecules is enabled and the system allowed to relax over 8 million simulation steps. Finally, data acquisition is carried out over 50 million simulation steps (which correspond to 125000 LJ-times).

To analyse the pseudo-FRET, the positions of the chromophores are tracked and the number of close encounters between donors (species A) and acceptors (species B) is registered. A close encounter happens when the distance between a donor and an acceptor turns less than 3 monomer diameters (or 1.5 chromophore-diameters). It is further necessary to implement a frequency cut-off, or latency-time between two subsequent close encounters between chromophores, to avoid the unphysical situation of chromophores exhibiting FRET continuously while staying immobilized in close contact. The choice of this latency time affects the shape of the FRET-curve in Figure 4 (main text, green curve) and yields closest fits with a cut-off about 20000 LJ-times. The choices of grafting density and co-solvent-monomer interaction strength affect the brush heights, and therefore these simulation parameters have been selected in order to fit the experimental results as closely as possible.

Time-dependent density functional theory (TD-DFT) calculations. All density functional theory (DFT) and time-dependent DFT (TD-DFT) calculations were carried out using the Gaussian 16, Revision C.01 quantum chemistry package.[4] Structures of NBD and Rhod B (Figure S6) were first optimised in the gas phase using the M06-2X DFT functional and 6-311+G(d,p) basis set. For the calculation of optical properties in solvent, several combinations of basis sets and DFT functionals were tested, with the B3LYP/6-31++G(d,p) level of theory providing the best agreement with experimental values and therefore used in all subsequent steps. For the TD-DFT calculations, implicit solvent (water, methanol, and N-hexane) was treated using the Polarizable Continuum Model (PCM)[5] with the SMD variation for solvent atomic radii.[6] Calculation of optical properties (absorbance and emission wavelength and oscillator strength) proceeded in four steps: (1) geometry optimisation of the ground state in implicit solvent, (2) TD-DFT calculation of absorbance, (3) TD-DFT excited state geometry optimisation, and (4) TD-DFT calculation of emission. For each geometry optimisation, a frequency

calculation was performed to ensure the resultant structure was at a stationary point on the potential energy surface. The results are reported in Table S1.

Results and Discussion

^{1}H NMR spectra:

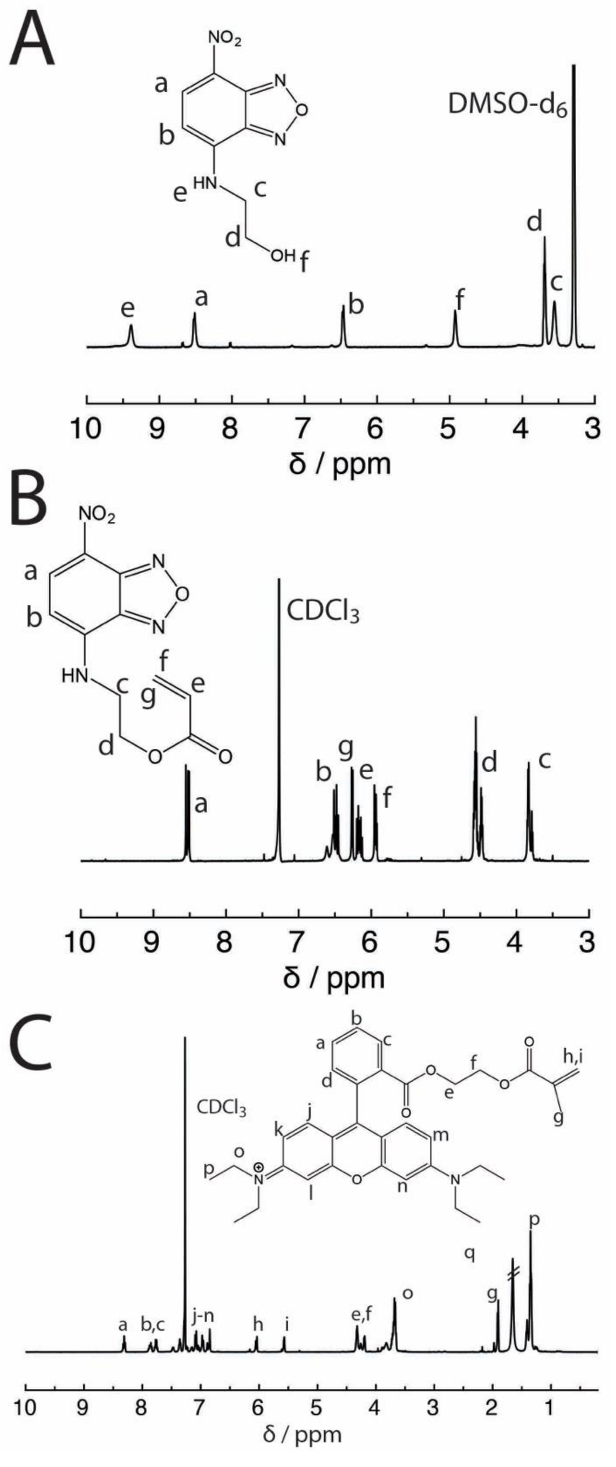

Figure S1. ^{1}H NMR spectra of 2-((7-nitrobenzo[*c*][1,2,5]oxadiazol-4-yl)amino)ethan-1-ol in DMSO-d_6 (A), NBD-AA in $CDCl_3$ (B), and Rhod B-HEMA in $CDCl_3$ (C).

Product appearance:

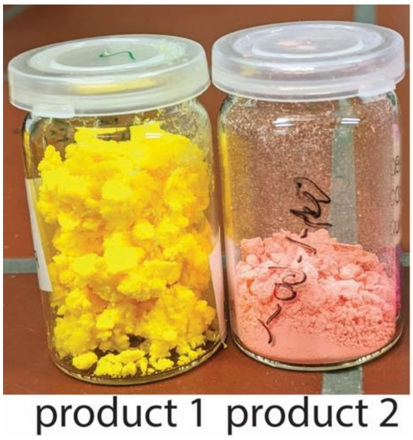

Figure S2. Digital photographs of product **1** and product **2**, highlighting the successful incorporation of dyes within the polymers.

CLSM:

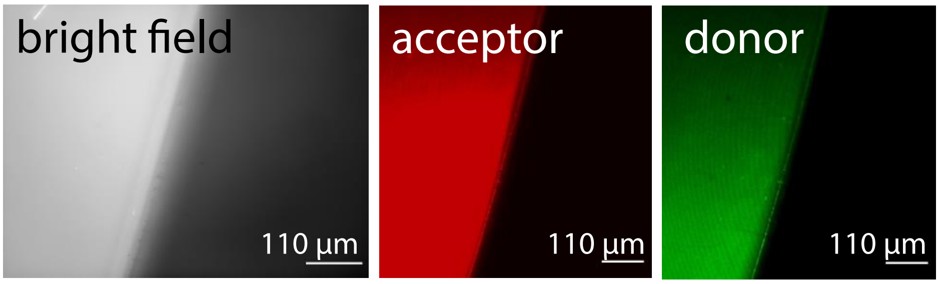

Figure S3. Bright field and CLSM images of the edge the polymer brush surface (product **3** of the main text). The acceptor channel is taken between 560 and 700 nm, with λ_{exc} = 543 nm, while the donor channel is taken between 490 and 560 nm, with λ_{exc} = 458 nm.

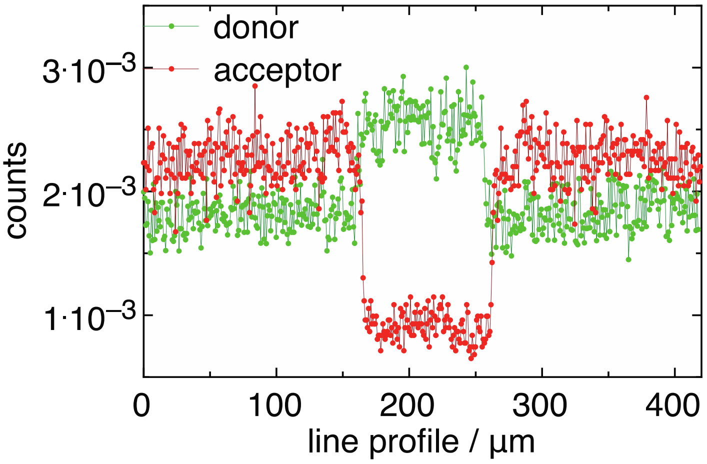

Figure S4. Normalised line profiles from across the acceptor photobleached areas of Figure 1H and I of the main text.

Fluorescence spectra:

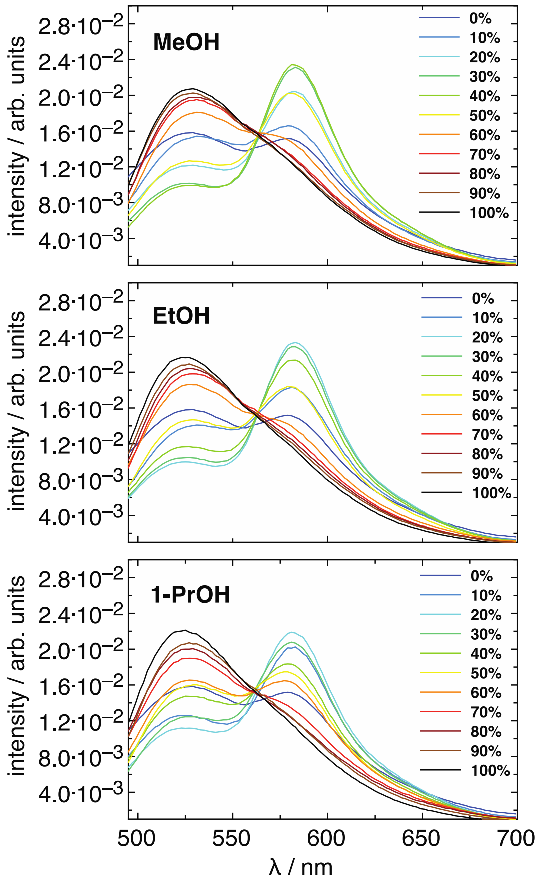

Figure S5. Fluorescence emission spectra of the free polymer (product **2**) in different water/alcohol mixtures. For all spectra λ_{exc} = 454 nm, and the polymer was at a concentration of 0.2 µg mL^{-1}. All measurements were performed at 24 °C.

TD-DFT:

Table S1. Calculated wavelengths, λ, and oscillator strengths, O_s, for the absorbance and emission of the NBD and Rhod B analogues (NBD-A and Rhod B-A, respectively) in different solvents.

Monomer		Water		MeOH	
		λ / nm	O_s	λ / nm	O_s
NBD-A	Absorbance	475.37	0.6620	471.92	0.6096
	Emission	534.17	0.4168	530.36	0.4081
Rhod B-A	Absorbance	549.81	1.3788	540.32	1.3843
	Emission	574.48	1.3212	560.70	1.3392

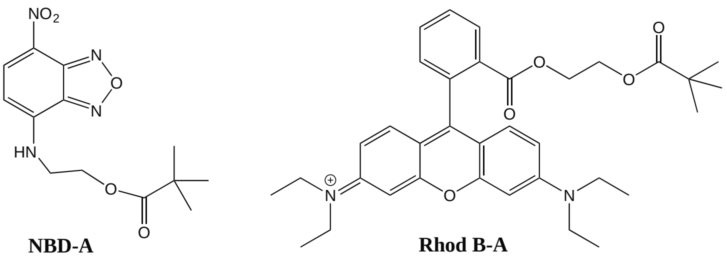

NBD-A **Rhod B-A**

Figure S7. Molecular structure of the NBD-A and Rhod B-A monomers used in TDDFT calculations of the fluorescence oscillator strengths, O_s.

Author Contributions

Q.A.B. conceptualised the project and performed the majority of experimental studies and wrote the manuscript. H.Y. performed ellipsometry measurements, H.M. performed simulation studies, and A.J.C. performed density functional theory calculations. Q.A.B., J.U.S., P.U. and A.F. supervised the studies. All authors reviewed the manuscript.

[1] H. Yong, S. Rauch, K. J. Eichhorn, P. Uhlmann, A. Fery, J. U. Sommer, *Materials (Basel)* **2018**, *11*.
[2] K. Kremer, G. S. Grest, *J. Chem. Phys.* **1990**, *92*, 5057.
[3] S. Plimpton, *J. Comput. Phys.* **1995**, *117*, 1-19.
[4] M. J. Frisch, G. W. Trucks, H. B. Schlegel, G. E. Scuseria, M. A. Robb, J. R. Cheeseman, G. Scalmani, V. Barone, G. A. Petersson, H. Nakatsuji, X. Li, M. Caricato, A. V. Marenich, J. Bloino, B. G. Janesko, R. Gomperts, B. Mennucci, H. P. Hratchian, J. V. Ortiz, A. F. Izmaylov, J. L. Sonnenberg, D. Williams-Young, F. Ding, F. Lipparini, F. Egidi, J. Goings, B. Peng, A. Petrone, T. Henderson, D. Ranasinghe, V. G. Zakrzewski, J. Gao, N. Rega, G. Zheng, W. Liang, M. Hada, M. Ehara, K. Toyota, R. Fukuda, J. Hasegawa, M. Ishida, T. Nakajima, Y. Honda, O. Kitao, H. Nakai, T. Vreven, K. Throssell, J. A. M. Jr., J. E. Peralta, F. Ogliaro, M. J. Bearpark, J. J. Heyd, E. N. Brothers, K. N. Kudin, V. N. Staroverov, T. A. Keith, R. Kobayashi, J. Normand, K. Raghavachari, A. P. Rendell, J. C. Burant, S. S. Iyengar, J. Tomasi, M. Cossi, J. M. Millam, M. Klene, C. Adamo, R. Cammi, J. W. Ochterski, R. L. Martin, K. Morokuma, O. Farkas, J. B. Foresman, D. J. Fox, Gaussian 16 rev. C.01, **2009**.
[5] G. Scalmani, M. J. Frisch, *J. Chem. Phys.* **2010**, *132*, 114110.
[6] A. V. Marenich, C. J. Cramer, D. G. Truhlar, *J. Phys. Chem. B* **2009**, *113*, 6378-6396.
